# The ABCs of finding a good antibody: How to find a good antibody, validate it, and publish meaningful data

**DOI:** 10.12688/f1000research.11774.1

**Published:** 2017-06-08

**Authors:** Poulomi Acharya, Anna Quinlan, Veronique Neumeister

**Affiliations:** 1 Bio-Rad Laboratories, Inc., Hercules, CA, 94547, USA; 2 Department of Pathology, Yale University School of Medicine, New Haven, CT, 06510, USA

**Keywords:** antibody validation, western blotting antibodies, immunohistochemistry, primary antibodies, antibody quality, reproducibility crisis

## Abstract

Finding an antibody that works for a specific application can be a difficult task. Hundreds of vendors offer millions of antibodies, but the quality of these products and available validation information varies greatly. In addition, several studies have called into question the reliability of published data as the primary metric for assessing antibody quality. We briefly discuss the antibody quality problem and provide best practice guidelines for selecting and validating an antibody, as well as for publishing data generated using antibodies.

## Introduction

Antibodies are widely used for applications that range from flow cytometry and immunohistochemistry to western blotting and ELISA. Even though antibodies are central to basic research as well as drug development and diagnostics, quality concerns remain high, and finding an antibody that works well for a specific application is a formidable challenge.

One source of the antibody quality problem is that it is not easy to generate a high-performing antibody. Production of monoclonal and polyclonal antibodies relies on an animal’s immune response, which is unpredictable and can vary from animal to animal even when they have the same genetic background. Some proteins do not elicit a strong immune response, others are too immunogenic, and yet others share too much homology with non-target proteins to yield a highly specific antibody. As part of the Human Protein Atlas project, Berglund *et al* quantified their antibody production success rate; 49% of their 9,000 internally generated antibodies failed validation ( Berglund *et al.*, 2008).

The Berglund study also highlights a second source of the antibody problem: commercially available antibodies have similar failure rates. This confirmed what many already knew to be true; just because an antibody is commercially available does not ensure its quality. However, the study also points to the solution. Failure rates among the 51 represented vendors ranged from 0 to 100%, suggesting that proper validation and quality control can allow vendors to provide high quality reagents.

The high failure rate of commercially available antibodies identified by Berglund and authors of similar studies is concerning because time and money are wasted. An estimated US$800 million are wasted annually on poorly performing antibodies and US$350 million are lost in biomedical research because published results cannot be replicated, with bad antibodies the likely culprit in many cases ( Bradbury & Plückthun, 2015).

For example, several years of research from multiple laboratories suggested that erythropoietin activates the erythropoietin receptor (EpoR) in tumor cells; a follow-up study, however, showed that only one of the four EpoR antibodies used in these studies detected EpoR and none of the four antibodies were suitable for immunohistochemistry ( Elliott *et al.*, 2006). Similarly, Prassas and Diamandis spent two years and $500,000 investigating CUZD1, a potential biomarker for pancreatic cancer, using an ELISA assay that turned out to recognize CA125 instead ( Prassas & Diamandis, 2014). Cases such as these have led some to suggest that irreproducibility should carry with it greater consequences, such as a requirement for academic institutions to return some or all of the grant money used to fund studies that prove irreproducible ( Rosenblatt, 2016).

The EpoR and CUZD1 examples not only demonstrate the devastating effect poorly performing antibodies can have on a research program, they also emphasize a third component of the antibody problem: the lack of enforced standards for antibody validation. Vendors frequently show cropped blots, share validation data that lack appropriate controls, or use large amounts of purified protein or cell lines overexpressing proteins of interest instead of physiologically relevant samples as positive controls. Such practices make it impossible to accurately assess an antibody’s performance. Similarly, journals have historically not provided guidelines for publishing validation data for antibody based assays.

Here we have compiled and share recommendations for (I) how to scrutinize available antibodies pre-purchase, (II) how to validate antibodies post-purchase, and (III) what information to include in publications to ensure that antibody quality and results can be evaluated by the reader.

### I. Selecting the best antibodies to test for your application

The first step to finding an antibody can be the most daunting — identifying antibodies that could work for your application. Product information and validation data can be difficult to decipher, and with hundreds of vendors to choose from it becomes difficult to know when a search has been exhaustive. The following guidelines are meant to simplify the process of identifying high-quality antibodies.

***1.***  ***Use search engines to find and compare available antibodies.***
  Search engines, such as those available through Biocompare, SelectScience, UniProt, or NCBI, allow you to find and in some instances even compare antibodies from many different vendors. This saves valuable time that is otherwise spent visiting each vendor’s website, and allows you to extend your search to vendors you may not be familiar with.
***2.***  ***Match the antibody type to your application.***
  Antibodies fall into three classes, polyclonal, monoclonal, and recombinant, each with distinct advantages and disadvantages.
  A polyclonal antibody is a mixture of antibodies that all recognize different epitopes of the protein of interest. This makes these antibodies well-suited for proteins that may have posttranslational modifications or heterogeneity in structure or sequence, proteins present at low concentrations, or applications that require fast binding to a protein of interest. Because polyclonal antibodies are generated in animals, they show relatively high batch-to-batch variability and are thus a poor choice for long-running studies that require repurchasing of the antibody, or for applications that have low tolerance for variability. If your experiments have low tolerance for variability, but only polyclonal antibodies are available, ask the vendor to provide antibodies from only a single lot.
  Monoclonal antibodies are generated by a single B-cell line and thus recognize only a single epitope of a protein of interest. This makes these antibodies highly specific and results generated with them more reproducible. Their high specificity makes monoclonals an ideal choice for immunohistochemistry applications, and the ability to generate immortal B-cell hybridomas ensures greater batch-to-batch homogeneity. Because these antibodies recognize a single epitope they can be more challenging to work with when looking at low-abundance proteins or proteins that show variability, such as those with posttranslational modifications, in the epitope recognized by the antibody.
  One caveat of monoclonal antibodies is that immortal B-cell hybridomas are not as eternal as their name implies; cell lines can die, not recover from frozen stocks, or even lose their antibody gene. Thus, for applications that have no tolerance for variability, recombinant antibodies are recommended. These custom synthetic antibodies provide an unlimited supply of identical antibodies, removing any batch-to-batch variability. Recombinant antibodies can be engineered to bind an epitope of choice with much higher affinity than that obtained *in vivo*. Because large libraries can be screened in a high-throughput manner, antibodies can be generated that distinguish similar compounds and bind their ligands only under desired conditions, such as a specific pH.
  The high reproducibility and entirely animal-free production process has led the pharmaceutical industry to adopt recombinant antibodies as their preferred tool. Many academics, on the other hand, understandably consider recombinant antibodies a last resort due to their higher cost. However, particularly for long-term studies, recombinant antibodies should be seriously considered due to their batch-to-batch consistency and their guaranteed continuity of availability without any dependence on animal immunization.
***3.***  ***Buy from companies that will work with you.***
  Choose a vendor who is willing to help you troubleshoot if an antibody does not perform as expected. If a vendor is unable or refuses to do so it may be a sign that they did not validate the antibody or that they are selling antibodies purchased from another vendor without additional quality control or the expertise to advise customers. Avoid vendors who provide only generic troubleshooting advice as this will be of little use if problems are encountered, and it suggests a lack of technical expertise. Regardless of the reason for not helping customers troubleshoot, a vendor’s inability to do so will leave you without technical support should it be needed.
  Also be careful of what may at first glance seem like generous exchange or return programs — letting customers test multiple antibodies for their target of interest can indicate poor quality and turns you, the customer, into an antibody testing tool.
***4.***  ***Look for antibodies with complete validation data.***
  Be wary of incomplete validation data; this is often a sign that an antibody is of poor quality and/or that the vendor will be able to do little to help you troubleshoot if the antibody does not perform as expected. Look for vendors who show the entire western blot image, provide detailed validation protocols, and validate their antibodies using multiple biologically relevant sample types or tissues. Not only do multiple sample types speak to the ability of an antibody to detect varying levels of expression, they often also reveal sample types that can be used as negative controls for your experiments.
  It is also important to carefully scrutinize a vendor’s validation data. If the positive control is merely purified protein, keep in mind that the specificity of the antibody remains unknown, since you are not looking at a complex biological sample. Make sure the vendor specifies how much protein was loaded and compare this amount to that expected in your sample. If your protein of interest is present in much lower amounts, you may still be able to use the antibody for some applications by enriching for your protein of interest through fractionation or IP.
***5.***  ***Select antibodies that have been validated for your application.***
  Whenever possible choose an antibody that is recommended by the vendor for your species and application. If such an antibody does not exist, contact the antibody vendor; in some cases the antibody may have failed validation for your application, while in other cases the vendor may not have tested it. You can also look to validation data in published studies to evaluate antibody performance. If no validation data are available for your application, choose a trusted vendor rather than one who simply states that antibodies have been validated for all applications. If you must use an antibody for non-recommended applications, be prepared to rigorously validate the antibody and to optimize vendor-suggested protocols for your specific experimental conditions.
***6.***  ***Check to ensure additives are compatible with your application.***
  Vendors often include additives that stabilize and extend the shelf life of antibodies. For most applications this is unproblematic, but there are some notable exceptions. For example, sodium azide can interfere with HRP-conjugated antibodies, antibody conjugation, and staining of live samples. Similarly BSA should not be added to antibodies that you will conjugate because it competes with the antibody for your label and can reduce conjugation efficiency. Another common additive, glycerol, lowers the freezing point to below -20°C, preventing freeze-thaw damage at -20°C because the antibody does not freeze. This cryoprotection does not extend to -80°C; at this temperature even antibodies stored in glycerol will freeze and will thus be subject to freeze-thaw damage ( Johnson, 2012). If you are adding glycerol to an antibody yourself, be sure to use sterile glycerol as it is easily contaminated with bacteria.
  When an antibody is not available without the interfering additive you may have to take steps to remove the additive or work with the vendor to see whether they can supply the antibody without the interfering additive. Additives can be removed through dialysis or by using commercially available kits. Keep in mind that these steps can reduce the antibody’s concentration and impact its performance.
***7.***  ***Review publications, but carefully scrutinize antibody data and references.***
  Journals like *Nature* and *JBC* are now starting to enforce guidelines for publishing antibody data, but this was not true in the past. When reviewing the literature, trust an antibody cited in a publication only if appropriate positive and negative controls are included. A new antibody should have validation data as well. When references are provided in place of validation data confirm that the authors of the original study performed and published the required validation experiments. If validation data are not presented in the original study, contact the authors to request this information. If authors cannot provide validation data, use the antibody only with the highest degree of caution and be sure to thoroughly validate the antibody before using it for your experiments.
  Focus your literature search on studies similar to yours. An antibody that performs well for flow cytometry may not be a good choice for immunoprecipitation, and host specificity can vary greatly. As you review the literature, be wary of antibodies that show discrepancies, such as an antibody detecting proteins of different molecular weights or showing different protein expression patterns in the same tissue types in different studies. If an antibody detects a protein with an unexpected molecular weight, look for controls that validate that the protein detected is actually the target protein.
  If authors show cropped western blots, contact them to request the full blot before you purchase the antibody. And if you struggle with a published antibody, don’t hesitate to contact the authors as they can often provide valuable troubleshooting information.

### II. Validating antibodies for your application

Once you have selected two to five promising candidates, the time-consuming process of validating these antibodies for your application begins. The temptation to skip this process, especially when an antibody vendor has not provided extensive validation data, should be resisted. However, if a vendor has provided extensive validation data, including data for your sample or a closely related sample type and application, there may be no need to test multiple antibodies.

Nevertheless, always test antibodies yourself on your sample, regardless of the antibodies’ source and validation state. Validation data provided by vendors do not always reflect the current antibody lot, antibodies may perform differently in your hands, and although it doesn’t occur frequently, mistakes do happen during antibody production and processing. For example, a research laboratory at an academic center recently encountered unexpected specificity issues within the same lot of an antibody that had been validated and used successfully over an extended period of time. The source of this problem was a packaging error.

***1.***  ***Optimize protocols for your specific applications.***
  Always optimize protocols and antibody dilutions and report final concentrations used. It is important to know the concentration of an antibody as dilutions are meaningful only when the stock concentration is known. Contact the vendor, as many will provide this information when queried. If the vendor has tested the antibody using physiologically relevant samples and provides detailed validation protocols, use their experimental conditions as a starting point. This can help considerably to reduce effort and time spent testing for the optimal conditions.
  If antibody-based protein evaluation is performed in a quantitative manner, signal-to-noise ratio and dynamic range are two of the most critical objective parameters to define the best antibody concentration for a given assay. Using too much antibody can yield nonspecific results, and too little can lead to no data or false-negative results. Based on the antibody application, the critical steps should be outlined and the experiment should have proper controls in place to make sure there are no or minimal artifacts. Optimizing assay conditions by conventional DAB/IHC should also be performed using a range of antibody concentrations.
  Pay attention to protein-specific antigen retrieval methods, as it is best to follow the vendor’s recommendations when optimizing antibody concentration. If the assay does not perform as expected, different retrieval methods may yield better results. Note that as you alter retrieval methods the optimal antibody concentration might need to be adjusted as well.
***2.***  ***Test each antibody for specificity, sensitivity, and reproducibility.***
  When assessing specificity, sensitivity, and reproducibility it is key to keep your intended application in mind. Will you be looking at native proteins or denatured proteins, a complex biological sample or a purified protein? These considerations will allow you to set meaningful performance criteria that an antibody must meet. Whenever possible, set quantitative quality control criteria rather than using qualitative measures that are often less reproducible and stringent ( Ramos *et al.*, 2016).
  The specificity of an antibody can be assessed by comparing its performance in cell lines with and without the target protein; signal in knock-out cell lines can be attributed to unspecific binding ( Bordeaux *et al.*, 2010). When knock-out cell lines are not readily available, RNAi can be used to knock down the protein of interest. If protein shows tissue-specific expression patterns, another easy way to assess specificity is by using samples known to express and not express the protein of interest.
  Sensitivity can be assessed by using protein-specific index arrays that contain sample and/or cell lines with varying but known amounts of target protein ( Carvajal-Hausdorf *et al.*, 2015; Welsh *et al.*, 2011). A simpler method for assessing the sensitivity of an antibody is to spike a sample that does not express the protein of interest with known amounts of purified protein.
  To assess reproducibility, run your validated antibody on 20 – 40 tissue samples, either as whole tissue sections or represented on a tissue microarray (TMA) for IHC. For western blotting, it is key to run replicates of lysates generated from the same batch of cells. Irrespective of the application, run your experiment in triplicate, using the same lot of antibody on different days and by different operators. In addition, use antibodies from different lots to compare lot-to-lot reproducibility. If you have previously used the antibody or trust published data generated using the antibody, compare your results to those data.
  Comparing antibodies from different vendors targeting the same protein adds further value to validation and reproducibility assessments. It is, however, important to consider that antibodies raised against different epitopes of the same protein can yield significantly different results, depending on how accessible a given epitope is in a sample of interest.
  Perform your validation experiments using the same buffers, sample types, and experimental conditions that will be used for your final experiments. An antibody validated in one buffer system will not necessarily perform similarly in another.
  Keep in mind that purified protein is sufficient to benchmark the target protein’s molecular weight in your sample, but it does not allow you to draw conclusions about specificity because purified protein is not a complex biological sample. Purified protein also does not allow you to determine the sensitivity or dynamic range of an antibody unless a dilution curve is set up to establish these parameters. Also keep in mind that purified proteins are often tagged, which changes their molecular weight. To facilitate antibody validation, whenever possible choose a vendor that provides a physiologically relevant positive control sample rather than a purified protein.
***3.***  ***Run controls with every experiment.***
  Every experiment should include a positive and negative control to assess antibody performance, ideally a set of samples with variable expression levels of the protein of interest. Protein-specific TMAs consisting of tissue samples and/or a set of cell lines can also be run alongside the experiments for quality control and reproducibility purposes. Arrays of cell lines with a range of expression levels and target-specific test TMAs can be purchased from a number of vendors. When a protein of interest is not expressed in immortalized cell lines or is expressed only transiently during a specific developmental stage, tissue samples may have to be used to validate an antibody’s performance.
  Knock-out or knock-down cells or samples known to not express the protein of interest are also frequently used as negative controls, especially since techniques like CRISPR and siRNA have simplified generation of such cell lines. Samples overexpressing the protein of interest, or even purified recombinant proteins, are commonly used as positive controls. However, results from such experiments are not always physiologically relevant, as knockdowns or knockouts can cause compensatory changes in cellular physiology. One way to avoid these pitfalls is to test samples with varying, known endogenous expression levels of the target protein. When researchers are working with freshly isolated primary cells or tissue samples, this becomes particularly important since over expression or knock-down validation is not always feasible.
  Depending on your application, additional controls should be included. For example, every quantitative western blot should include a housekeeping protein loading control unless you are performing total protein normalization (TPN), and every ELISA should include a standard curve. In both cases, make sure that your signal is within the assays’ dynamic range. When using TPN, be aware that this method detects proteins by interacting with tryptophans (Trp). If the total amount of Trp in your sample is altered by your experimental treatment, TPN will no longer serve as a reliable control.
***4.***  ***Retest antibodies before using them with an important sample.***
  Antibodies have limited shelf lives and are often shared resources in a laboratory. It is therefore wise to retest your antibody before performing a critical experiment. This does not need to be full validation; in these cases a quick experiment with relevant controls under previously established conditions is sufficient to ensure that an antibody is still performing as expected.
***5.***  ***Store antibodies as recommended by the vendor.***
  Carefully review vendor recommendations and store antibodies accordingly. Write the date of first use on the vial to track antibody usage and do not store working dilutions in buffer for later use because this can affect stability; as you dilute your antibody you are also diluting stabilizers added by the vendor. If an antibody has been stored for a long time or has expired, it is best to use it only with caution. Validation experiments should be repeated and working concentrations may need to be adjusted as antibody stability decreases over time. If you have altered vendor storage conditions by, for example, removing additives or stabilizers, the antibody shelf life can decrease significantly. It is thus advisable to carefully mark any alterations in storage or formulation so that both current and future users are aware of these changes.
***6.***  ***Train all new lab personnel.***
  Take the time to familiarize new lab members with proper antibody etiquette. Ensure that they understand the importance of antibody validation, proper controls, and agreed upon best practices.

### III. Publishing meaningful antibody data

Most journals do not specify reporting criteria for the publication of antibody-generated data. This is highly problematic because many scientists turn to previously published data to inform not only their antibody choice but also the direction of their research. As the reproducibility debate is gaining momentum, more journals are defining stricter reporting criteria ( Fosang & Colbran, 2015; http://www.nature.com/authors/policies/image.html). Until these criteria are universally enforced, it falls to the scientific community to implement minimum guidelines both in their own publications and when participating in the peer review process.

***1.***  ***Provide complete antibody information.***
  The full antibody name, vendor, lot number, and antibody concentration and dilution, and incubation time should be provided. If a new in-house antibody is used, include information about how the antibody was generated.
***2.***  ***Always include positive and negative controls in published data.***
  All antibody-generated data should include positive and negative controls, as well as all additional controls required for your particular application (loading controls for western blots, standard curves for ELISAs, etc.). Not including these controls makes published data uninterpretable.
***3.***  ***Include validation data for all new antibodies.***
  When using non-established antibodies or established antibodies for a new application, validation data that determine antibody specificity, sensitivity, and reproducibility should be presented. This information can be included in supplementary data, but should not be missing from the published study. Without this crucial information conclusions drawn from presented experiments are difficult to evaluate.
***4.***  ***Present complete data and describe all quantitative methods.***
  Do not crop western blots or splice lanes from different blots into a single image. If lanes need to be cropped out of a blot, crop lines should be clearly indicated. All quantitation using antibodies should be described carefully in the methods or supplementary materials, including how signal intensity was measured, linearity of the assay was determined, and signal was normalized for quantitation.

## Conclusions

The antibody quality problem is well documented in the literature and can no longer be ignored. With growing discussion and awareness vendors and scientists alike must be held to higher validation and reporting standards. We have summarized the above minimum best practice guidelines in Table 1 in the hope that they will simplify the antibody search, serve as a starting point for further conversation, and improve the quality of antibody data published until strict antibody reporting standards are agreed upon and universally enforced.

**Table 1.** Antibody best-practice guidelines.

Pre-Purchase	Post-Purchase	Publication
Compare antibodies from different vendors Select antibody type (monoclonal, polyclonal, recombinant) that matches your application needs Pick antibodies validated for your application Choose vendors that will work with you Look for complete validation data Ensure additives are compatible with your application Review publications critically	Optimize protocols for your application Test all antibodies for sensitivity, specificity, and reproducibility Retest antibodies before using them on an important sample Run positive and negative controls with all experiments Store antibodies as recommended Train new lab personnel on proper antibody etiquette	Provide complete antibody information (antibody name, vendor, catalog number, lot number, dilution) Include proper controls in all published data Include validation data for new antibodies Present complete data and describe quantitative methods

## References

[ref-1] Berglund L Björling E Oksvold P: A genecentric Human Protein Atlas for expression profiles based on antibodies. *Mol Cell Proteomics.* 2008;7(10):2019–27. 10.1074/mcp.R800013-MCP200 18669619

[ref-2] Bordeaux J Welsh A Agarwal S: Antibody validation. *Biotechniques.* 2010;48(3):197–209. 10.2144/000113382 20359301 PMC3891910

[ref-3] Bradbury A Plückthun A: Reproducibility: Standardize antibodies used in research. *Nature.* 2015;518(7537):27–9. 10.1038/518027a 25652980

[ref-4] Carvajal-Hausdorf DE Schalper KA Pusztai L: Measurement of Domain-Specific HER2 (ERBB2) Expression May Classify Benefit From Trastuzumab in Breast Cancer. *J Natl Cancer Inst.* 2015;107(8): pii: djv136. 10.1093/jnci/djv136 25991002 PMC4554192

[ref-5] Elliott S Busse L Bass MB: Anti-Epo receptor antibodies do not predict Epo receptor expression. *Blood.* 2006;107(5):1892–5. 10.1182/blood-2005-10-4066 16249375

[ref-6] Fosang AJ Colbran RJ: Transparency Is the Key to Quality. *J Biol Chem.* 2015;290(50):29692–4. 10.1074/jbc.E115.000002 26657753 PMC4705984

[ref-7] Johnson M: Antibody Shelf Life/How to Store Antibodies. *Mater Methods.* 2012;2:120 10.13070/mm.en.2.120

[ref-8] Prassas I Diamandis EP: Translational researchers beware! Unreliable commercial immunoassays (ELISAs) can jeopardize your research. *Clin Chem Lab Med.* 2014;52(6):765–6. 10.1515/cclm-2013-1078 24497227

[ref-9] Ramos P Leahy A Pino I: Antibody Cross-Reactivity Testing Using the HuProt™ Human Proteome Microarray.2014 Reference Source

[ref-10] Rosenblatt M: An incentive-based approach for improving data reproducibility. *Sci Transl Med.* 2016;8(336):336ed5. 10.1126/scitranslmed.aaf5003 27122610

[ref-11] Welsh AW Moeder CB Kumar S: Standardization of estrogen receptor measurement in breast cancer suggests false-negative results are a function of threshold intensity rather than percentage of positive cells. *J Clin Oncol.* 2011;29(22):2978–84. 10.1200/JCO.2010.32.9706 21709197 PMC3157961

