# Supplementary material for: The ABCs of finding a good antibody: How to find a good antibody, validate it, and publish meaningful data
Source: F1000Res. 2017 Jun 8;6:851. [Version 1] doi: 10.12688/f1000research.11774.1 (PMC5499787; doi:10.12688/f1000research.11774.1)
Supplement: Peer review history

# Peer review: Referee response for version 1

**DOI:** 10.5256/f1000research.12720.r23340

**Published:** 2017-08-14

**Authors:** Andrew D. Chalmers

**Affiliations:** 1 CiteAb and The Department of Biology and Biochemistry, University of Bath, Bath, UK

The opinion article by Poulomi Acharya and colleagues is an interesting commentary on three important areas that relate to antibody use, how to select, validate and report antibody use. 

 I think it provides a valuable contribution to discussion in this field and makes many valuable points on the topic. 

 I would like to suggest a number of minor changes/corrections that should be considered if/when the article is revised.

1. The authors state in the abstract that “in addition, several studies have called into question the reliability of published data as the primary metric for assessing antibody quality”. I think if the authors are going to make this statement in the abstract they should specifically return to it in the article and discuss it more widely. Which studies are they and what were the conclusions. What other metrics are available and do the authors believe these should these be used instead or with published data? 
  My own opinion is that there are clear cases where peer reviewed published antibody results have turned out not to be reliable and the authors are right to raise them and warn researchers. I also believe that supplier validation is in many cases a very good source of information, reviews can also provide value, but I would argue that peer reviewed published results, when available and looked at critically, are still the best source of information on antibody quality, short of validating the antibody in your own laboratory. 
2. I would of course agree with the authors that researchers should use search engines to help identify possible candidate antibodies (Page 2). The validation data for these antibodies can then be investigated and a final choice made. However, the search engines proposed omit several well used ones such as CiteAb (I am obviously biased), but also Antibodypedia and PabMabs and others. The three sites mentioned rank by citations or reviews and complement Biocompare and Select Science which I believe rank on a financial basis. I am also not aware of how UniProt and NCBI can be used as antibody search engines so some explanation might be helpful.
3. It is not true that all monoclonal antibodies are highly specific (Page 2), a recent study showed this for antibodies against the oestrogen receptor ^1^.
4. Recombinant monoclonals can potentially show batch-to-batch variability caused by changes in the manufacturing process, so I think it is more accurate to say that they should have the least batch-to-batch variability, rather than saying “removing any batch to batch variability” (Page 3).
5. I think the authors are right to the stress the need to select antibodies, where possible, that have been validated for the application of interest (Page 3; final paragraph). I wonder if the authors could also make more of the need to try and find antibodies validated for the tissue and cell type of interest?
6. They are also right to point out that comparing antibodies from different vendors can add further value to the validation (page 4), but it might be worth stressing that due to cross selling researchers need to be careful to make sure that the antibodies are actually different.
7. In section III, number 1. The authors have omitted the catalogue code as part of the information that should be listed. They do put this in Table 1. I think this should be added and would also suggest the clone number and any conjugate can also provide valuable information and should in an ideal world be included.
8. Two of the authors work for an antibody supplier and this should be declared.

I have read this submission. I believe that I have an appropriate level of expertise to confirm that it is of an acceptable scientific standard, however I have significant reservations, as outlined above.

## References

[1] AnderssonSSundbergMPristovsekNIbrahimAJonssonPKatonaBClaussonCMZiebaARamströmMSöderbergOWilliamsCAsplundA: Insufficient antibody validation challenges oestrogen receptor beta research. *Nat Commun*.2017;8: 10.1038/ncomms15840 15840 10.1038/ncomms15840 28643774PMC5501969

# Peer review: Referee response for version 1

**DOI:** 10.5256/f1000research.12720.r23765

**Published:** 2017-06-30

**Authors:** C. Glenn Begley

**Affiliations:** 1 BioCurate Pty Ltd, Parkville, VIC, Australia

Given that Bio-Rad Laboratories is a supplier of antibodies to the scientific community, the two authors who are employed by Bio-Rad Laboratories could be perceived as having a ‘competing interest’. This should be declared as such.

  

1. My biggest concern with this paper is what might be interpreted to be an over-reliance upon published studies to support the use of antibodies for particular indications. For example:
  (i)The statement that “Vendors frequently show cropped blots, share validation data that lack appropriate controls, or use large amounts of purified protein or cell lines overexpressing proteins of interest instead of physiologically relevant samples as positive controls” is correct.
  But this is further compounded by investigators who continue the same practices and further contaminate the literature by reporting results that purport to support the use of a particular antibody by not disclosing its lack of specificity.
  (ii) The comment “Their high specificity makes monoclonals an ideal choice for immunohistochemistry applications” is correct. However it is worth adding the caveat that simply because these are monoclonal antibodies, does not guarantee that they will be suitable for immunohistochemistry. The control experiments are still required to validate their utility.
  (iii) It is strongly recommended that the statement “You can also look to validation data in published studies to evaluate antibody performance” be modified. One should be extremely careful about relying upon the published literature as a source of confirmation for an antibody.
  It is very rare that published studies validate an antibody for the purpose for which it is applied. It is unfortunately much more common that antibodies that should not be used immunohistochemistry or flow cytometry are used for that purpose. Once the first paper is published, subsequent investigators simply cite that paper as evidence of ‘validation’ without any confirmatory studies. This is a major problem for careful investigators.
  (iv) “When reviewing the literature, trust an antibody cited in a publication only if appropriate positive and negative controls are included.” Because most publications typically only show a tiny ‘window’ of a gel, I recommend adding the phrase “and for western blots and immunoprecipitation experiments, only if the entire gel is shown”.
2. It may be worth commenting that different vendors sell the same antibody but with a different name and lot number. Thus while an investigator is purchasing antibodies that appear to be different, they might actually be the identical antibody. Researchers should at least be aware that is currently occurring.
3. With respect to “Publishing meaningful antibody data”, I suggest the Authors add a comment that
  (i) All westerns and IPs should have size standards shown (in addition to showing the complete gel).
  (ii) Subjective assessment of IHC (for example counting the number of metastases in the lung) should be performed by blinded investigators.
  (iii)For flow cytometry experiments, “outlier points” should not be removed.
  (iv)Experiments should be repeated – investigators should resist publishing a single positive western result, or analysing a single IHC sample as “typical”.
4. “IP”, “DAB”, “IHC” should be defined when they occur in the text.

I have read this submission. I believe that I have an appropriate level of expertise to confirm that it is of an acceptable scientific standard.

# Peer review: Referee response for version 1

**DOI:** 10.5256/f1000research.12720.r23624

**Published:** 2017-06-26

**Authors:** Alison H. Banham

**Affiliations:** 1 Nuffield Division of Clinical Laboratory Sciences (NDCLS), Radcliffe Department of Medicine (RDM), John Radcliffe Hospital, University of Oxford, Oxford, UK

Antibody validation and its contribution to scientific reproducibility is a topical area. While there are several recent reviews on the subject each brings a slightly different perspective that adds value. This is true of the current article, which provides some very helpful and practical advice. The article also highlights the key role of dialogue between antibody vendors and end users, between academic laboratories and the importance of a partnership within the scientific community to improve the standards of antibody validation and publishing antibody-based data.

 While commercially available antibodies have the advantage of being easily accessible it would be worth mentioning the published literature earlier than section 7 as a resource for finding antibodies. In some instances the best antibody may be produced by an academic laboratory and might not be commercially available.

 The section on recombinant antibodies gives the impression that these reagents are always generated by library screening, “without any dependence on animal immunization”. It would be helpful to clarify that any antibodies, including classical monoclonal antibodies derived from hybridoma cell lines, can be produced in a recombinant format by isolating and cloning their immunoglobulin genes. Indeed recombinant therapeutic antibodies used by the pharmaceutical industry are commonly derived from classical monoclonal antibodies and efforts are underway to convert many monoclonal antibodies used as research tools into a recombinant format to ensure their longevity.

 While the authors indicate they have no competing interests it would be worthwhile for full transparency to declare that two of the authors work for a commercial antibody vendor, particularly as the article makes strong recommendations regarding criteria for vendor selection.

I have read this submission. I believe that I have an appropriate level of expertise to confirm that it is of an acceptable scientific standard.

# Peer review: Referee response for version 1

**DOI:** 10.5256/f1000research.12720.r23764

**Published:** 2017-06-26

**Authors:** Steven Elliott

**Affiliations:** 1 Elliott Consulting, Newbury Park, CA, USA; 2 Amgen Inc (retired), Thousand Oaks, CA, USA

This paper is a welcome addition to the community because it identifies a significant problem (antibody nonspecificity) and provides guidelines for improvement. While there are helpful recommendations in this submission, including how to do experiments and recommendations on finding antibodies, it does not describe a complete and proper plan. The authors indicate that antibodies should be validated but do not provide enough detailed information on how this should be done nor the criteria expected of a properly validated antibody. There is also no detailed guidance on how to validate all procedures, reagents and thereby select proper controls. In this regard checklists would be helpful.  For example, staining intensity must match expression level, a band on a gel must be the correct size, negative controls must be included, sensitivity of the antibody should be determined and sufficient to detect the target in the sample of interest etc. Red-flags that would invalidate an antibody should also be described. Preferably a second antibody should be used for cross-validation and it must give the same pattern of staining.

 Specific comments:

 Page 2 paragraph 5(left). All of the antibodies in Elliott *et al., *2006 ^1^ detected EpoR, however the sensitivity of all were low with poor specificity. One of the 4 antibodies (M-20- Santa Cruz Inc) was initially thought to be useful for westerns because it passed some tests. However M-20 was later invalidated because it detected a correctly sized protein thought to be EpoR that turned out to be a non-EpoR protein. (see Elliott *et al., *2013 ^2^).

 Page 2 paragraph 6 (right). Polyclonal antibodies are not necessarily more sensitive. In fact because of the elevated noise with polyclonals, they can have a poor signal to noise ratio.

 Page 2 paragraph 7 (right). High specificity and reproducibility are related to affinity and the nature of the binding site (epitope), not to monoclonal antibodies vs polyclonal *per se*.

 Page 3 paragraph 4 (right): The premise that an investigator can trust any other lab or publication misses what should be the main point of the paper. The ultimate responsibility must belong to the end user. Even under the best circumstances it is impossible to fully evaluate validation done by others. For example, how does one know if only select data is shown or whether experiments were repeated or reproducible etc.

 Page 4 paragraph 2(left). Few manufacturers do “extensive” validation. Vendors frequently only show limited and selective (best) data with little description of the validation of reagents and controls. 

 Page 4 paragraph 2(left). A discussion on the limitations of RNAi is warranted. Like antibodies, RNAi can also give misleading data due to non specific knockdown of the presumed target protein.

 Page 5 last paragraph (right). A discussion of what are appropriate positive and negative “controls” is missing.

I have read this submission. I believe that I have an appropriate level of expertise to confirm that it is of an acceptable scientific standard, however I have significant reservations, as outlined above.

## References

[1] ElliottSBusseLBassMBLuHSarosiISinclairAMSpahrCUmMVanGBegleyCG: Anti-Epo receptor antibodies do not predict Epo receptor expression. *Blood*.2006;107(5) : 10.1182/blood-2005-10-4066 1892-5 10.1182/blood-2005-10-4066 16249375

[2] ElliottSSwiftSBusseLScullySVanGRossiJJohnsonC: Epo receptors are not detectable in primary human tumor tissue samples. *PLoS One*.2013;8(7) : 10.1371/journal.pone.0068083 e68083 10.1371/journal.pone.0068083 23861852PMC3701640
